# Anti-disturbance control design of Exoskeleton Knee robotic system for rehabilitative care

**DOI:** 10.1016/j.heliyon.2024.e28911

**Published:** 2024-03-31

**Authors:** Ayad Q. Al-Dujailii, Alaq F. Hasan, Amjad J. Humaidi, Ammar Al-Jodah

**Affiliations:** a Electrical Engineering Technical College, Middle Technical University, Baghdad, 10022, Iraq; b Technical Engineering College, Middle Technical University, Baghdad, Iraq; c Control and Systems Engineering Department, University of Technology, Baghdad, Iraq; d University of Western Australia, Perth, WA 6907, Australia

**Keywords:** Exoskeleton knee, Nonlinear extended state observer (NESO), Linear extended state observer (LESO), Particle swarm optimization (PSO), ADRC

## Abstract

In this study, Active Disturbance Rejection Control (ADRC) has been designed for motion control of knee-joint based on exoskeleton medical robot. The extended state observer (ESO) is the main part of ADRC structure, which is responsible for estimating both actual states and system uncertainties. The proposed control scheme has adopted two versions of observers as disturbance estimators: linear extended state observer (LESO) and nonlinear extended state observer (NESO). The efficacy of proposed ADRC is strongly related to the performance of used ESO. As such, a comparison study has been conducted to evaluate the performance of two ADRCs in terms of disturbance-rejection capability and robustness to variation in system parameters under two version of ESO (LSO and NLESO). In order to enhance the dynamic performance of ADRC, Particle Swarm Optimization (PSO) algorithm has been used to optimally tune the design parameters of control scheme. To show the effectiveness of proposed LESO-based ADRC and NLESO-based ADRC, numerical simulation have been conducted. The proposed controllers have tested for an uncertain exoskeleton-knee system, where a 20% change in parameters was permitted over their nominal values. The results indicate that the ADRC algorithm based on LESO outperforms the one based on NESO in terms of disturbances rejection ability and robustness to parameters’ variations.

## 1 Introduction

The number of people who suffers from strokes that consequently lead to long-term disability is alarmingly high. According to the WHO (World Health Organization), out of the 15 million people who are experiencing strokes worldwide, approximately 5 million people suffer from long-lasting disabilities. It has been reported that the primary causes of acute and long-life disabilities are due to neurological injuries such as heart attack, brain strokes, and SCI (spinal cord injury). Therefore, it is vital to acknowledge the impact that these disabilities can have on those affected, as well as their families and communities [1].

Patients experiencing musculoskeletal injuries and neurological disorders may face a loss of mobility and independence. As a result, numerous physical symptoms can occur, including muscle weakness, reduced sensation either partially or completely, weakened cognitive abilities, and less alertness. Consequently, these challenges significantly impact the patients’ well-being. Medical reports have shown that a potential remedy exists for such individuals through a dedication to physical rehabilitation and therapy sessions. Promising outcomes can be achieved when patients are directed to engage in specific tasks through long-term and repetitive exercises [1,2].

These exercises are manually performed under the help and supervision of physicians. As such, this approach can incur significant costs due to physician involvement and the requirement of their direct guidance on the exercises that are being executed [2,3]. Actuated bio-engineering exoskeletons can be a promising solution that reduces the dependence on human assistance during time-consuming rehabilitation exercises. Moreover, these exoskeletons have the capacity to assist several patients instantaneously, as they can easily perform physical movements repetitively without exhaustion. Therefore, resulting in less workload on therapists. Furthermore, these devices utilize specialized sensors for monitoring, recording, measuring, and quantifying the progress in treatment for patients under tests. Which in turn facilitates the tracking of several measurements, such as angular position, velocity, exerted torques, and others. These measurements can easily be incorporated into extensive reports for each case, which then be accessed by physicians to interpret, and analyze results, and decide the course of action for the next treatment. It was confirmed in clinical trials that robotic exoskeletons exhibited significant efficiency and effectiveness in rehabilitation treatments [2,3].

One of the crucial steps to consider when pursuing rehabilitation using exoskeleton-like robots is the customization of the device to fit the specific human joint and its range of motion. It is imperative that the assisting devices are carefully attached and coupled to the patient's body to ensure that the wearer can comfortably tolerate the ergonomic motion. This consideration is vital for lower-limb orthoses, where the exoskeleton-like robot aims to restore as many movements as possible, ultimately helping patients to regain their independence and autonomy [2,3].

There are two interconnected links which constitutes the exoskeleton robot fixed at of knee joint. The first link is securely tied to the thigh of patient, while the other link is fixed with the shank of leg. A direct current (DC) motor is used to actuate the assistive robot with necessary torque required to rotate the shank around the knee joint. Control theory plays a vital role in ensuring the smooth and precise movement of the disabled knee. However, the exact modeling and precise parameter acquisition of the exoskeleton pose significant challenges for the control systems, especially considering that the device is used by multiple individuals with varying physical properties. Thus, it is essential to develop control schemes that are adaptive, robust, and accurate, as they play a crucial role in this application.

According to the type of actuator, the Exoskeleton medical robots can be actuated either by electric or hydraulic actuators. In hydraulic-actuated robots, there is a limitation in device motion. In addition, the hydraulic-actuated medical robots are complicated and nonlinear due to the presence of various valves; especially the switching control valve which is responsible for changing the direction of actuating signals. As such, nonlinear control approaches have been applied to control such type of medical robot like sliding mode control, synergetic control and adaptive control. The literature has been enriched by many works which addressed the use of hydraulic actuators for rehabilitation system [4,5].

The prosthetic knees can be divided into two types: computerized and mechanical knees. There is the single-axis, multi-axis, and polycentric knees in mechanical knees. The polycentric knee may have 4 axes of rotation (four bar knees) or seven axes of rotation (seven bar knees). In polycentric knees (Multi axial knees), the prosthesis can be shorten in swing, which in turn reduces the risk of falling and tripping [6].

The following literature review and discussions are focused on the related studies that address the control theory of the exoskeleton lower-limb system. H. Rifaï et al. [7] have proposed an adaptive control scheme for controlling the lower-limb of disabled patient at the level of knee joint to replace the physician role in rehabilitation. The control and adaptive laws for proposed model reference adaptive control (MRAC) has been designed and developed based on stability analysis using Lyapunov theorem to achieve bounded actuating torque signal. K. Sherwani et al. [8] have used adaptive Robust Integral of Sign Error (RISE) controller to achieve high precision of trajectory tracking for knee-Exoskeleton device. The robust controller has been designed to cope with nonlinearities due to modeling errors, parameter uncertainties and applied disturbances. Based on Lyapunov theory, semi-global asymptotic stability has been proven for exoskeleton-human system and the proposed adaptive RISE controller showed better robustness characteristics as compared to conventional RISE controller. A. Aljuboury et al. [9] presented comparison study of three controllers based on MRAC approach for angular position control of assistive exoskeleton device for knee joint. The first MRAC is based on nonlinear observer, the second MRAC utilized adaptive disturbance observer, while the last controller was based on conventional MRAC. The numerical results showed that the MRAC based on nonlinear observer had the best tracking and robustness performances as compared to classical MRAC and MRAC based on adaptive observer. N. A. Alawad et al. [10] has proposed active disturbance rejection control for controlling the lower-limbs (upper and lower legs). The proposed controller has addressed the cross-coupling problem between the lower and upper legs. The study conducted a comparison study between the proposed ADRC and PD controller. The numerical simulations showed the superior of proposed controller over the conventional control. G. Ding et al. [11] applied APSMC (adaptive proxy-based sliding mode control) for controlling the shank-orthosis system. A comparison study has been made between the proposed APSMC, the conventional PSMC (proxy-based sliding mode control) and the adaptive PD controller. The tracking accuracy and robustness characteristics can be considerably improved by the proposed APSMC. S. Mefoued et al. [12] proposed robust controller based on HOSMC (high sliding mode controller) to control the knee-joint for rehabilitation purposes. As compared to PID (Proportional-Integral-Derivative) controller, the HOSMC showed better error-convergence speed, accuracy and robustness against external uncertainties. W. Zhao and A. Song [13] proposed PSMC (proxy-based sliding mode control) for motion control of PMA-based knee exoskeleton device. In terms of accuracy and trajectory tracking, the performance of PSMC outperforms that based on conventional controllers. M. Ajayi et al. [14] proposed bounded control scheme based on observer to help in conducting repeated exercises for defected lower-limbs of disabled patients for rehabilitation purposes. The amount of actuated torque is given to the lower limb according to the estimated values of angular position and velocity using high gain observer. S. Mahdi et al. [15] proposed synergetic-based adaptive controller for angular position of exoskeleton robot to rotate the shank around the knee-joint. The device is devoted to assist disabled persons to perform controlled exercises prescribed by physicians. The controller has been designed to cope with uncertainties and load changes due to change in the weight of legs for different tested patients. H. Rifaï et al. [16] have presented L1-Adaptive control design for actuating rehabilitation robot at knee level to track desired trajectory which assists in recovering of disabled persons. The classical L1-adaptive controller has been augmented with nonlinear controller to compensate the time lag due to the presence of filter. As compared to conventional, the augmented L1-adaptive controller results in better trajectory tracking performance. S. Mefoued and D. Belkhiat [17] used observer-based controller to drive knee-exoskeleton robot fixed at the lower-limb of patient. The sliding mode observer has been used to estimate the parameters of the exoskeleton system and to provide the angular velocity. As compared to PID controller, the observer-based controller showed better robustness performance against external disturbances and system's parameters. C. –F. Chen et al. [18] applied fast terminal SMC-based Active disturbance rejection control (ADRC) to enhance the performance of trajectory tracking for lower-limb exoskeleton device. The FTSMC can compensate the disturbance and give fast convergence. The ADRC has the advantage of reducing the chattering level due to SMC. As compared to PID controller, the FTSMC-based ADRC has better tracking performance. J. Wang et al. [19] presented observer-based finite-time controller for trajectory tracking control of PAM-actuated exoskeleton robot to improve the movement ability of disabled lower-limbs (hip and knee joints). The lumped uncertainties and angular velocities are estimated based on extended state observer (ESO). High-precision and fast tracking performance are obtained with proposed controller. A. Chevalier et al. [20] designed FOPI (Fractional-Order Proportional-Integral) controller to regulate the shank motion around the joint of human knee by controlling the actuated torque. The approximation of transfer function for FOPI controller to integer-order transfer function with finite dimension is the key to this study. The FOPI controller has been tested for different types of knees and compared to IOPI (integer-order PI) controller. The results showed the superiority of proposed controller over the conventional PI controller in terms of robustness and tracking performance. S. Kaur et al. [21] proposed internal model FOPID controller to regulate the motion of shank around the joint of human knee. Some design parameters of POPID controller are tuned based on try-and-error procedure, while the others are tuned using the concept of IMC (internal mode control). Compared to other fractional order control methods, the proposed FOPID showed better robustness characteristics and tracking performance. S. Mefoued et al. [22] presented robust controller based on SOSMC (second-order sliding mode control) approach to control the human limbs in order to reinforce or restore the weakness or loss of functions in these limbs. The controller had been designed to cope with cross-coupling, nonlinearities and uncertainties of orthosis. In terms of accuracy, tracking errors and robustness against uncertain perturbation, the proposed SOSMC outperforms the conventional controllers. S. Mefoued [23] developed adaptive intelligent control based on neural network using Multi-layer Perceptron Neural Network (MLPNN) to actuate the lower limb (orthosis). The proposed adaptive controller does not need dynamic model of orthosis. As compared to PID controller, the proposed MLPNN showed better characteristics in terms of bounded actuated torque and robustness. N. A. Al-Awad et al. [24] have presented different schemes of ADRC approach to actuate the lower-limb for rehabilitation purpose. The proposed ADRC schemes have taken different structures and connections and utilized fractional order theory in the design of PID controller and Extended Stated Observers (ESO). Y. Zhang et al. [25] have proposed controller based on input-output data acquisition of exoskeleton robot fixed at knee-joint. The controller is devoted to help restoring and reinforcing the weak and injured limbs by presenting systematic controlled exercises with prescribed trajectory. The prior torque and discrete SMC have been employed to improve the robustness and tracking accuracy of controller. The key feature of proposed controller is that there is no need for the exoskeleton model. In Ref. [26], Mefoued and Belkhiat developed a robust control methodology for a knee-exoskeleton, employing an observer-based sliding mode controller to assist individuals with restricted knee mobility. The proposed controller showcased superior precision and robustness in a comparative study with a PID controller. Aole et al. [27] presented improved version of ADRC for controlling a 2-DOF LLRRE (Lower Limb Robotic Rehabilitation Exoskeleton). The improved ADRC showed high capability to reject noise and external disturbances and proved strong robustness against variations in system parameters.

In spite that the previous works have addressed good robust controllers, but there is few works have considered output feedback control for exoskeleton systems at the level of knee joint. However, these observer-based controllers have not addressed the cancellation of uncertainties based on its observation. In this study, the observer is devoted to estimate both actual states and uncertainty of the system and feedback control is responsible for rejection of these uncertainties. As such, the Active Disturbance Rejection Control (ADRC) has been presented for this purpose. The ADRC is an effective control strategy capable of handling complex uncertainties inherent in nonlinear systems. This method, pioneered by Han in the late 1980s and 1990s, is capable of managing a mix of unmolded dynamics and disturbances. A vital component of the ADRC is the Extended State Observer (ESO), which is used to estimate the total uncertainties. This control strategy has been implemented in a variety of control problems, and it has exhibited its remarkable PID-like attributes, which include operating independently of the mathematical model and irrespective of control accuracy [[28], [29], [30], [31], [32], [33], [34], [35]].

## 2 Modeling of Exoskeleton-Knee Assistive system

**Fig. 1:**
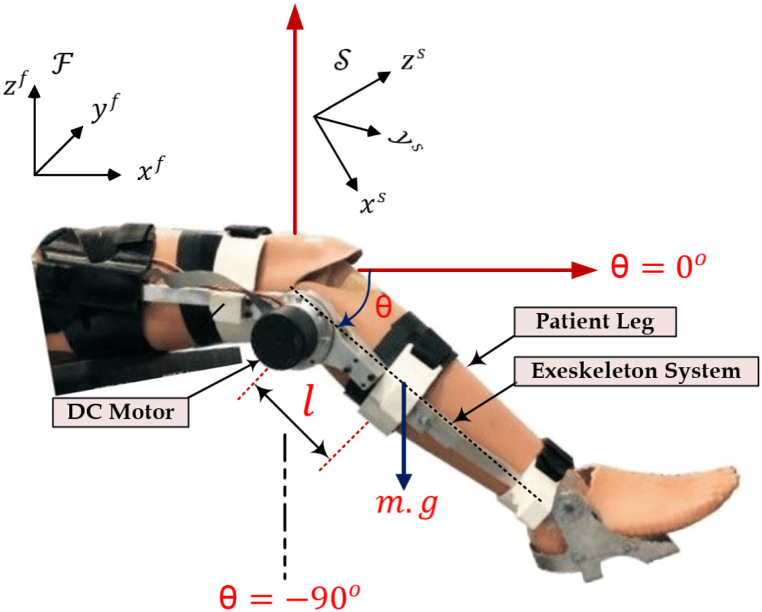
Geometric representation of exoskeleton knee.

Fig. 1 shows the details of the exoskeleton aided-knee system. There two links constituting the exoskeleton aided-knee system. The first one is stationary and tied to the thigh of patient, while the second link is movable in circular motion and it attached to the leg shank. The system is supported by a dc motor to actuate the movable part (shank-part) around the knee joint. The motor produces the specified torque to enable the rotation of the leg precisely, which help a disable knee. The range of motion of the exoskeleton was restricted to $\left[0^{o}-90^{o}\right]$, a full extension is at $0^{o}$, while a resting posture is at $90^{o}$.

Two coordinate frames were used, the first one is the global frame F: (${\vec{x}}^{f}$, ${\vec{y}}^{f}$, ${\vec{z}}^{f}$), and it is stationary. The second frame is S: (${\vec{x}}^{s}$, ${\vec{y}}^{s}$, ${\vec{z}}^{s}$), is a moving frame that is attached to the knee and rotate at an angle θ. It is worth noting that ${\vec{y}}^{f}$ and ${\vec{y}}^{s}$ will coincides at some point during the rotational motion of S. Based on this kinematics, the motion of the knee joint can be described by its angle θ and its velocity $\dot{\theta }$. In this work, Lagrangian method was utilized to model the both the exoskeleton and the human leg. Initially, the human leg and exoskeleton device were considered as a coupled system and described by [5,24]

(1) $$l_{i}=E_{ki}-E_{gi}$$

In this context, i, can be either 1: the human leg system, or 2: the exoskeleton system. $E_{gi}$ and $E_{ki}$ respectively stand for the gravitational and kinetic energies of these system components.

(2) $$E_{ki}=\frac{1}{2}J_{i}\;{\dot{\theta }}^{2}$$

where, $J_{i}$ denotes the inertia for the system's elements, specifically referring to the human leg and the exoskeleton.

(3) $$E_{gi}=m_{i}.g.\;l_{i}\;.\left(1-\sin\;\theta \right)$$

where $m_{i}$ stands for the mass of the components, which are the human leg and the exoskeleton system, g denotes the acceleration due to gravity. Lastly, $l_{i}$ corresponds to the distance between the knee joint and center of gravity.

The dynamic model of the coupled system can be derived using Euler-Lagrange differential equation as follows

(4) $$J_{i}\ddot{\theta }=m_{i}.g.\;l_{i}\;.\;\cos\;\theta -\tau_{exti}$$

where, $\tau_{exti}$ denotes the entire external torque that's being exerted on the system, and it is $\tau_{exti}=\tau_{fi}+\tau_{i}$ where $\tau_{i}$ is the input torque controlled by the actuator (DC motor), while $\tau_{fi}$ is a friction torque repressed by:

(5) $$\tau_{fi}=-f_{si\;}sgin\dot{\theta }-f_{vi}\;\dot{\theta }$$

here, $f_{si\;}$ and ${f_{v}}_{i}$ stands for the coefficients of solid friction and viscosity, respectively. The coupled exoskeleton system has a total dynamical model described by

(6) $$J\ddot{\theta }=-\tau_{g}\;\cos\;\theta -f_{s\;}sgin\dot{\theta }-f_{v}\;\dot{\theta }+\tau_{h}+\tau$$

where, $f_{s}=\sum_{i=1}^{2}f_{si\;}$, $f_{v}=\sum_{i=1}^{2}f_{vi}$, $\tau_{g}=\sum_{i=1}^{2}\tau_{gi}$, and $J=\sum_{i=1}^{2}J_{i}$. The variables θ, $\dot{\theta }$, and $\ddot{\theta }$, respectively, indicate the angular position, velocity, and acceleration of coupled system. J stands for the inertia of the combined system, which includes the exoskeleton and the human leg, $\tau_{g}$ is the torque due to gravity, τ is the control torque, and $\tau_{h}$ denotes the load torque resulting from the coupling of the system.

For the purpose of control system design, a state space system can be defined by renaming θ as $x_{1}$ and $\dot{\theta }$ as $x_{2}$, as follows

$${\dot{x}}_{1}=x_{2}$$

(7) $${\dot{x}}_{2}=\frac{1}{J}\;\left[\tau -f_{v}\;x_{2}-f_{s\;}sign\left(x_{2}\right)+\tau_{g}\;\cos\left(x_{1}\right)\right]$$

## 3 Design of active disturbance rejection controller

**Fig. 2:**
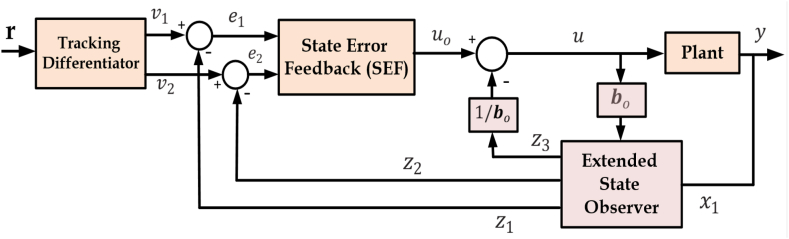
Structure of ADRC

Fig. 2 illustrates the structure of ADRC, which consists of three fundamental elements: the tracking differentiator (TD), the extended state observer (ESO), and the state error feedback controller. The TD function is to quickly obtain the tracking signal along with its differential from the input. The role of ESO is to predict the extended state, influenced by inherent uncertainties and external interferences [[36], [37], [38], [39]]. The final step towards achieving efficient control is the implementation of a non-linear combination of the state error to produce the control output.

### 3.1 Tracking differentiator (TD)

The TD plays a vital role in smoothing reference input corrupted by noise. The TD has another advantage, represented by reproducing the clean versions of reference signal and its derivative. The smoothed signals of TD are summed-up with corresponding feedback signals to provide error and change of error signals to the controller part. To develop the equations for TD, the following analysis has been conducted. Consider a double-integral plant described by [25,30].

(8) $$\left\{ \begin{array}{c} {\dot{x}}_{1}=x_{2}\\ {\dot{x}}_{2}=u \end{array}\right.$$

with |u|≤r*.* The time-optimal solution is

(9) $$u=-r\;sign\left(x_{1}-v+\frac{x_{2}\left|x_{2}\right|}{2r}\right)$$

where v represents the desired value for $x_{1}$. Accordingly, one can determine the desired profile by solving the following differential equations

(10) $$\left\{ \begin{array}{c} {\dot{v}}_{1}=v_{2}\\ {\dot{v}}_{2}=-r\;sign\left(v_{1}-v+\frac{v_{2}\left|v_{2}\right|}{2r}\right) \end{array}\right.$$

where, v is the desired trajectory and the state variables $v_{1}$ and $v_{2}$ represent, respectively, the smoothed tracking signal and its smoothed derivative of desired trajectory. In order to decelerate or quicken the transient profile, the design parameter r is used for this purpose based on specific physical limitations of each application.

In order to mitigate the chattering resulting from the sign-function indicated in Eq. (10), the ENTD (Enhanced Tracking Differentiator) is introduced by replacing sign-function by hyperbolic tangent-function. Using Enhanced Tracking Differentiator, one can extract the original version and its derivative from any reference signal corrupted by noise with high accuracy. The ENTD has filtering characteristics and it is capable of handling high nonlinearities due to noise [25],

(11) $$\left\{ \begin{array}{c} {\dot{v}}_{1}=v_{2}\\ {\dot{v}}_{2}=-r^{2}\;\tanh\left(\frac{\rho v_{1}-\left(1-\varepsilon \right)v}{\gamma }\right)-rv_{2} \end{array}\right.$$

where ρ,ε,γ and r are design parameters, and 0<ε<1, ρ>0, γ>0 and r>0.

Lemma 1 *Consider system given by* Eq. (11) *for* $\left|\frac{\rho v_{1}-\left(1-\varepsilon \right)v}{\gamma }\right|\ll 1$. *Both tracking error* $e_{t}\left(t\right)=v-\frac{\rho }{1-\varepsilon }v_{1}$ *and differentiation error* $e_{d}\left(t\right)=\dot{v}-\frac{\rho }{1-\varepsilon }v_{2}$ *is approximately zero for finite input signal*.

**Proof:**

Since $\frac{\rho v_{1}-\left(1-\varepsilon \right)v}{\gamma }\ll 1$, then $\tanh\left(\frac{\rho v_{1}-\left(1-\varepsilon \right)v}{\gamma }\right)\to \frac{\rho v_{1}-\left(1-\varepsilon \right)v}{\gamma }$ so that

(12) $$\left\{ \begin{array}{c} {\dot{v}}_{1}=v_{2}\\ {\dot{v}}_{2}=-r^{2}\left(\frac{\rho v_{1}-\left(1-\varepsilon \right)v}{\gamma }\right)-rv_{2} \end{array}\right.$$

Taking Laplace transform to Eq. (12), then:

(13) $$\left[\begin{array}{c} v_{1}\left(s\right)\\ v_{2}\left(s\right) \end{array}\right]=\left[\begin{array}{c} \frac{\frac{r^{2}\left(1-\varepsilon \right)}{\gamma }}{s^{2}+rs+\frac{r^{2}\rho }{\gamma }}\\ \frac{\frac{r^{2}\left(1-\varepsilon \right)s}{\gamma }}{s^{2}+rs+\frac{r^{2}\rho }{\gamma }} \end{array}\right]v\left(s\right)$$

The tracking phase error is

(14) $$e_{t}\left(t\right)=v-\frac{\rho }{1-\varepsilon }v_{1}$$

(15) $$E_{t}\left(s\right)=v\left(s\right)-\frac{\rho }{1-\varepsilon }v_{1}\left(s\right)$$

The tracking error transfer function with r is given by

(16) $$L_{t}\left(s\right)=\frac{E_{t}\left(s\right)}{v\left(s\right)}=\frac{s\left(s+r\right)}{s^{2}+rs+\frac{r^{2}\rho }{\gamma }}$$

Such that,

(17) $$l_{t}\left(\infty \right)=\lim_{s\to 0}sL_{t}\left(s\right)=0$$

The tracking phase differentiation error is

(18) $$e_{d}\left(t\right)=\dot{v}-\frac{\rho }{1-\varepsilon }v_{2}$$

(19) $$E_{d}\left(s\right)=sv\left(s\right)-\frac{\rho }{1-\varepsilon }v_{2}\left(s\right)$$

The T.F. (transfer function) of tracking error can be given by

(20) $$L_{d}\left(s\right)=\frac{E_{d}\left(s\right)}{v\left(s\right)}=\frac{s.\left(s+r\right)}{s^{2}+rs+r^{2}\rho /\gamma }$$

Such that,

(21) $$l_{d}\left(\infty \right)=\lim_{s\to 0}sL_{d}\left(s\right)=0$$

### 3.2 Extended state observer (ESO)

#### 3.2.1 Linear extended state observer (LESO)

Let us consider a nonlinear system subjected to external and internal perturbation and described by the following dynamic equation [36].

(22) $$\left\{ \begin{array}{c} {\dot{\zeta }}_{1}=x_{2}\\ {\dot{\zeta }}_{2}=f\left(\zeta ,w,t\right)+bu\\ y=\zeta_{1} \end{array}\right.$$

In this equation, u and y denote the input and output signals respectively, while f(ζ,w,t) represents an uncertain nonlinear system function that includes disturbances. The control gain is indicated by b. The new expanded state variable is $\zeta_{3}=f\left(\zeta ,w,t\right)+\Delta b\;u$, which incorporates the total disturbance of the system, ${\dot{\zeta }}_{3}=g\left(t\right)$ , then

(23) $$\left\{ \begin{array}{c} {\dot{\zeta }}_{1}=\zeta_{2}\\ {\dot{\zeta }}_{2}=\zeta_{3}+bu\\ \begin{array}{c} {\dot{\zeta }}_{3}=g\left(t\right)\\ y=\zeta_{1} \end{array} \end{array}\right.$$

The expanded state observer is

(24) $$\left\{ \begin{array}{c} {\dot{\hat{\zeta }}}_{1}={\hat{\zeta }}_{2}+\beta_{1}\;\left(\zeta_{1}-{\hat{\zeta }}_{1}\right)\\ {\dot{\hat{\zeta }}}_{2}={\hat{\zeta }}_{3}+b_{0}u+\beta_{2}\;\left(\zeta_{1}-{\hat{\zeta }}_{1}\right)\\ {\dot{\hat{\zeta }}}_{3}=\beta_{3}\;\left(\zeta_{1}-{\hat{\zeta }}_{1}\right) \end{array}\right.$$

where ${\hat{\zeta }}_{1}$, ${\hat{\zeta }}_{2}$, ${\hat{\zeta }}_{3}$ are the estimations of $\zeta_{1}$, $\zeta_{2}$, and $\zeta_{3}$. The stability of ESO is ensured by properly choosing the design parameters $\beta_{1}$, $\beta_{2}$ and $\beta_{3}$ according to $\left[\beta_{1},\beta_{2},\beta_{3}\right]=\left[3w_{o},3w_{o}^{2},w_{o}^{3}\right]$, where $w_{o}$ is the observer bandwidth.

Now, the state errors are defined as ${\tilde{\zeta }}_{1}=\zeta_{1}-{\hat{\zeta }}_{1}$, ${\tilde{\zeta }}_{2}=\zeta_{2}-{\hat{\zeta }}_{2}$ and ${\tilde{\zeta }}_{3}=\zeta_{3}-{\hat{\zeta }}_{3}$. Substituting in Eq. (24), results in the overall system:

(25) $$\left\{ \begin{array}{c} {\dot{\tilde{\zeta }}}_{1}={\dot{\zeta }}_{1}-{\dot{\hat{\zeta }}}_{1}=-3w_{o}\;{\tilde{\zeta }}_{1}+{\tilde{\zeta }}_{2}\\ {\dot{\tilde{\zeta }}}_{2}={\dot{\zeta }}_{2}-{\dot{\hat{\zeta }}}_{2}=-\;3w_{o}^{2}\;{\tilde{\zeta }}_{1}+{\tilde{\zeta }}_{3}\\ {\dot{\tilde{\zeta }}}_{3}={\dot{\zeta }}_{3}-{\dot{\hat{\zeta }}}_{3}=-w_{o}^{3}\;{\tilde{\zeta }}_{1}+{\dot{\zeta }}_{3} \end{array}\right.$$

The state error $\tilde{\zeta }={\left[{\tilde{\zeta }}_{1},{\tilde{\zeta }}_{2},{\tilde{\zeta }}_{3}\right]}^{T}$ is established to redefine Eq. (25) as:

$$\dot{\tilde{\zeta }}=A\tilde{\zeta }+B$$

(26) $$\left[\begin{array}{c} {\dot{\tilde{\zeta }}}_{1}\\ \begin{array}{c} {\dot{\tilde{\zeta }}}_{2}\\ {\dot{\tilde{\zeta }}}_{3} \end{array} \end{array}\right]=\left[\begin{array}{ccc} -3w_{o} & 1 & 0\\ -\;3w_{o}^{2} & 0 & 1\\ -w_{o}^{3} & 0 & 0 \end{array}\right]\left[\begin{array}{c} {\tilde{\zeta }}_{1}\\ \begin{array}{c} {\tilde{\zeta }}_{2}\\ {\tilde{\zeta }}_{3} \end{array} \end{array}\right]+\left[\begin{array}{c} 0\\ 0\\ {\dot{\zeta }}_{3} \end{array}\right]$$

As per Eq. (26), it is consistently feasible to choose $w_{o}$ in a manner that places the eigenvalues of A on the left-hand plane, enabling the identification of a positive definite matrix P,

(27) $$A^{T}P+PA=-Q$$

This is applicable for any Q>0 (positive definite matrix). Following this, a Lyapunov candidate can be defined

(28) $$V={\tilde{\zeta }}^{T}P\tilde{\zeta }$$

Differentiating Eq. (28) yields

$$\dot{V}={\dot{\tilde{\zeta }}}^{T}P\tilde{\zeta }+{\tilde{\zeta }}^{T}P\dot{\tilde{\zeta }}$$

$$\dot{V}={\left(A\tilde{\zeta }+B\right)}^{T}P\;\tilde{\zeta }+{\tilde{\zeta }}^{T}P\left(A\;\tilde{\zeta }+B\right)={\tilde{\zeta }}^{T}A^{T}P\tilde{\zeta }+{\tilde{\zeta }}^{T}PA\;\tilde{\zeta }+2B^{T}P\;\tilde{\zeta }$$

$$\dot{V}=-{\tilde{\zeta }}^{T}Q\tilde{\zeta }+2B^{T}P\tilde{\zeta }$$

$$\dot{V}\leq -\eta \left(Q\right){\left\|\tilde{\zeta }\right\|}^{2}+2\varepsilon \left\|P\right\|\left\|\tilde{\zeta }\right\|$$

Following an adequate duration, the norm of the state error $\tilde{\zeta }$ remains within a certain limit, given by

(29) $$\left\|\tilde{\zeta }\right\|\leq \frac{2\varepsilon \left\|P\right\|}{\eta \;\left(Q\right)}$$

here, η(Q) indicates the minimum eigenvalue of matrix Q.

Lemma 1 *Assuming a continuously differentiable function*Υ(t)*that fulfills the given conditions*:

(30) $$\sigma_{1}\leq \Upsilon \left(t\right)\leq \sigma_{2}$$

where $\sigma_{1}$ and $\sigma_{2}$ are positive constants. The derivative of Υ(t) is also restricted within limits. The estimation error ${\tilde{\zeta }}_{3}$ is confined, and based on Lemma 1, the derivative of ${\tilde{\zeta }}_{3}$ is also bounded

(31) $$\left|{\dot{\tilde{\zeta }}}_{3}\right|<L$$

where L represents a constant of real value.

#### 3.2.2 Nonlinear extended state observer (NESO)

Let ${\hat{\zeta }}_{i}$, where*,* i=1,2,…,n+1, denote the observed system states $\zeta_{i}$ as described in Eq. (23), and let e equal the difference between ${\hat{\zeta }}_{1}$ and $\zeta_{1}$, representing the observation errors. With this setup, the nonlinear extended state observer can be transformed as follows [37]:

(32) $$\left\{ \begin{array}{c} e={\hat{\zeta }}_{1}-\zeta_{1}\\ {\dot{\hat{\zeta }}}_{1}={\hat{\zeta }}_{2}-\beta_{4}e\\ \begin{array}{c} {\dot{\hat{\zeta }}}_{2}={\hat{\zeta }}_{3}-\beta_{5}\;fal\left(e,\alpha_{1},\delta \right)+b_{0}u\\ {\dot{\hat{\zeta }}}_{3}=-\beta_{6}\;fal\left(e,\alpha_{2},\delta \right) \end{array} \end{array}\right.$$

where $\beta_{4}$*,* $\beta_{5}$ and $\beta_{6}$ denote the observer gains. fal(.) is a continuous power function with linear segments in the vicinity of the origin, and it's expressed as follows:

(32) $$fal=\left\{ \begin{array}{c} e\;\delta^{\alpha -1}\;\left|e\right|\leq \delta \\ {\left|e\right|}^{\alpha }sgn\left(e\right)\;\left|e\right|>\delta \end{array}\right.$$

here, δ represents the threshold value between the linear and non-smooth (nonlinear) intervals, and α denotes the control parameter, and its value is between zero and one. If the absolute value of the error (|e|) is less than δ, then fal behave in a linear manner, and it is used to curb high-frequency oscillations induced by large gains in the sign function. Conversely, if |e| exceeds δ, then fal serves as a non-smooth function.

Remark.1 The system dynamics f(ζ,w,t) as represented in in Eq. (22) are continuously differentiable and restricted within certain limits. This assumption is practically reasonable for real-world systems. For instance, when the nonlinear characteristic f(ζ,w,t) represents the system's friction, the friction value is always constrained. Similarly, system disturbances and uncertainties are also not infinite in practical applications.

Remark.2 Due to the bounded nature of f(ζ,w,t), it can be inferred that $a\left(\zeta ,w,t\right)=f\left(\zeta ,w,t\right)+\left(b-b_{0}\right)u$ is also bounded. The nonlinear function fal(.) ensures that the observer states ${\hat{\zeta }}_{i}$ tends towards $\zeta_{i}$ when suitable parameters $\beta_{4}$, $\beta_{5}$ and $\beta_{6}$, are chosen. Additionally, and the observer errors converge to $\left|\zeta_{i}-{\hat{\zeta }}_{i}\right|\leq l_{i}$, where $l_{i}>0$ represent small positive values.

### 3.3 Nonlinear state error feedback (NSEF)

NSEF is constructed by combining the nonlinear state deviations associated with TD and ESO. This combination can be expressed as follows [38]:

(34) $$\left\{ \begin{array}{c} \begin{array}{c} e_{1}=v_{1}-{\hat{\zeta }}_{1}\\ e_{2}=v_{2}-{\hat{\zeta }}_{2} \end{array}\\ u_{o}=c_{1}\;fal\left(e_{1},\alpha_{3},\delta_{1}\right)+c_{2}\;fal\left(e_{2},\alpha_{3},\delta_{1}\right)\\ u=\frac{u_{o}-{\hat{\zeta }}_{3}}{b_{0}}\; \end{array}\right.$$

where, $c_{1},c_{2}$ are tunable scalars. fal(.) function efficiently mitigates the signal chattering and exhibits a beneficial filtering effect.

## 4 Numerical simulation

In this section, the effectiveness of the optimal ADRC algorithm utilizing LESO and the optimal ADRC algorithm utilizing NESO has been assessed through numerical simulations conducted using MATLAB package. The numerical simulations employed Ode 45 as the numerical solver. Furthermore, the simulations were conducted based on the following assumptions.

I. theta is restricted to a practical range of 0≤θ≤−90 degree.
II. The model of the EICoSI knee exoskeleton system incorporates the human effort as an external torque applied to the system. To simulate real-world conditions, a white-Gaussian noise with a mean value of zero and a standard deviation of 0.5 N m is introduced to the human torque.
III. The following parameters are used

$$J=0.4\;kg.m^{2},A=0.6\;N.m,B=1\;N.m.\sec.{rad}^{-1}\;\text{and}\;\tau_{g}=5\;N.m\text{.}$$

The optimization algorithm employing Particle Swarm Optimization (PSO) yields optimal design parameters for the ADRC algorithm based on LESO and the optimal ADRC algorithm based on NESO [38]. Additionally, other recent optimization techniques such as WOA (Whale-Optimization Algorithm) and GWO (Grey-Wolf Optimization) can be considered for optimizing the parameters of ADRC [39,40]. The cost function (fitness function) used to evaluate the solutions at every iteration is chosen on the basis of RMSE (Root Mean Square Error). The PSO algorithm is developed to conduct the solution such as to minimize the cost function in the global sense of the search space. The configuration of the design parameters-based PSO is presented in Table 1.

**Table 1:** Optimal setting of design parameters for LESO-based ADRC and NESO-based ADRC using PSO Algorithm.

LESO-based ADRC			NESO-based ADRC		
Block	Parameter	Value	Block	Parameter	Value
TD	r	270.0035	TD	r	270.0035
	ρ	0.8685		ρ	0.8685
	ε	0.1282		ε	0.1282
	γ	2.2246		γ	2.2246
LESO	$w_{0}$	539.3106	NESO	$\alpha_{1}$	0.1611
NSEF	$\alpha_{3}$	0.8823		$\alpha_{2}$	0.2334
	$\alpha_{4}$	0.5367		δ	0.0120
	$\delta_{1}$	697.8624		$\beta_{4}$	158.2496
	$b_{0}$	2.6949		$\beta_{5}$	413.3361
	$c_{1}$	797.7301		$\beta_{6}$	456.4765
	$c_{2}$	948.5469	NSEF	$\alpha_{3}$	0.8823
				$\alpha_{4}$	0.5367
				$\delta_{1}$	697.8624
				$b_{0}$	2.6949
				$c_{1}$	797.7301
				$c_{2}$	948.5469

This study considers two scenarios: one scenario assumes no uncertainty, while the other scenario incorporates uncertainty by assuming that the parameters of the system deviate by 20% from their nominal values.

**Fig. 3:**
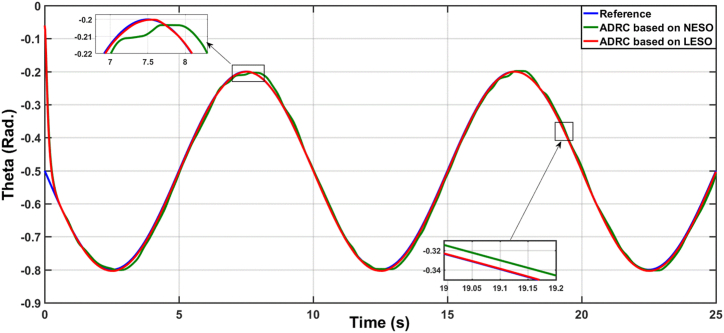
The responses of knee angular position due to ADRC algorithm based on LESO and NESO.

**Fig. 4:**
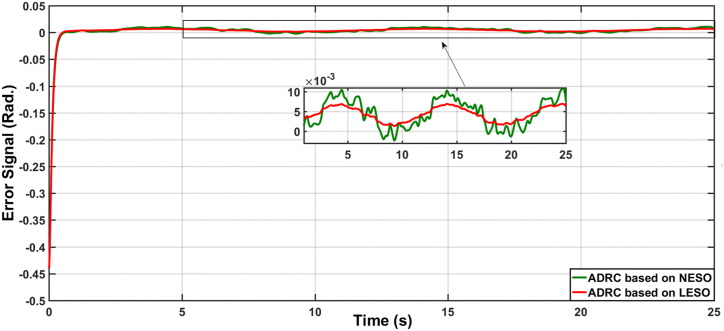
The error signal due to ADRC algorithm based on LESO and ADRC algorithm based on NESO.

Fig. 3 illustrates the variations of the theta angle in the uncertainty-free scenario using ADRC with both linear and nonlinear state observers, and Fig. 4 displays the error signal for the same. The performance of the proposed controllers is evaluated numerically as shown in Table 2. Metrics such as RMS (Root Mean Square), ISE (Integral of Squared Error), IAE (Integral of Absolute Error), and ITAE (Integral of Time multiplied by Absolute Error) were used. The results clearly demonstrate that the ADRC algorithm based on LESO significantly improves performance compared to the ADRC algorithm based on NESO.

**Table 2:** Transient parameters of the controlled system based on ADRC algorithm based on LESO and ADRC algorithm based on NESO under uncertainty-free case.

Controller Type	RMSE	ISE	IAE	ITAE
ADRC algorithm based on LESO	0.7901	15.383	110.321	693.742
ADRC algorithm based on NESO	0.8586	18.167	239.250	2241.45

**Fig. 5:**
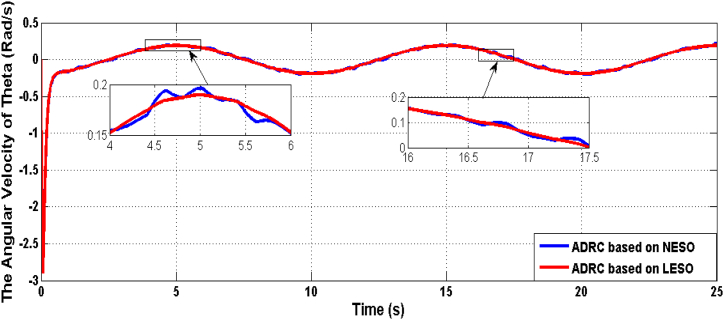
The rate of change of knee angular position due to ADRC based on LESO and NESO.

**Fig. 6:**
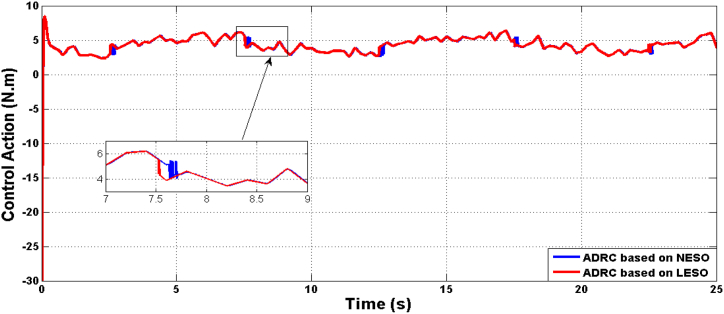
Shows the control effort due to both optimal ADRC based on LESO and NESO.

**Fig. 7:**
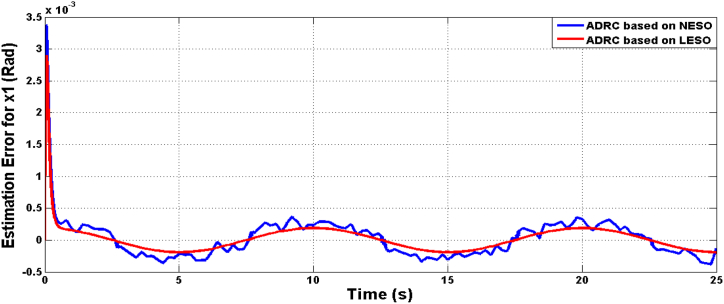
The estimation errors of knee angular position $x_{1}$ due to LESO and NESO.

**Fig. 8:**
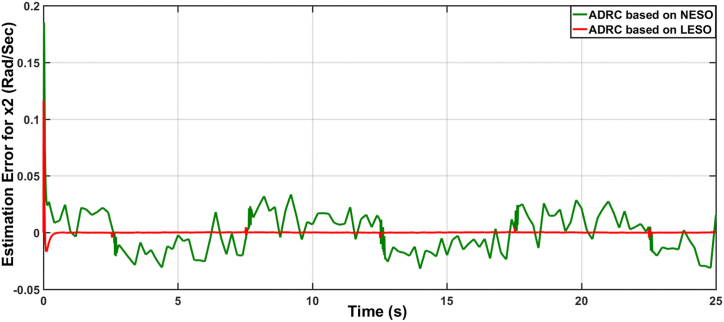
The estimation error of the time derivative for joint angle $x_{2}$ due to ADRC algorithm based on LESO and NESO.

Fig. 5 presents the rate of change of the theta angle resulting from both LESO and NESO. The control effort generated by the optimal ADRCs based on LESO and NESO is displayed in Fig. 6. Additionally, Fig. 7 and Fig. 8 depict the estimation errors of knee angular positions and their rate of change for the uncertainty-free scenario, with both proposed observers.

**Fig. 9:**
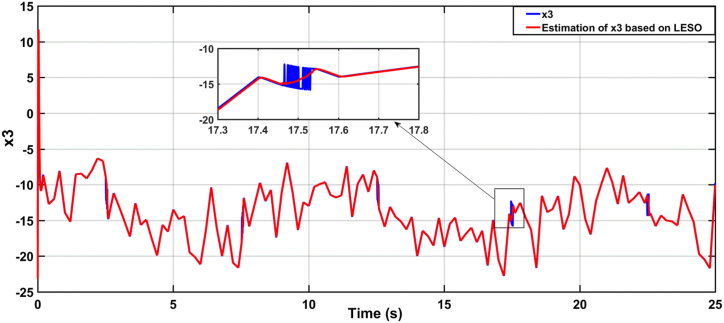
The behaviour of estimated state $x_{3}$ (lumped uncertainty) due to ADRC algorithm based on LESO.

**Fig. 10:**
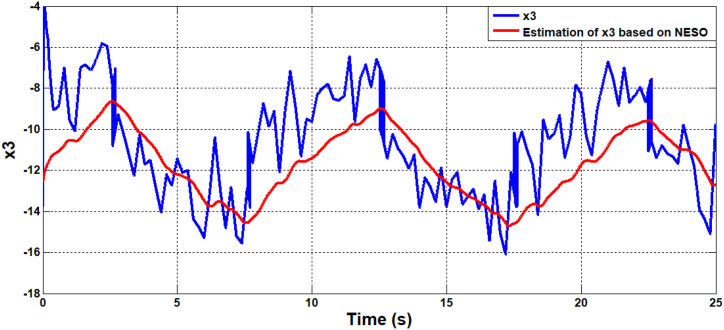
The behaviour of estimated state $x_{3}$ (lumped uncertainty) due to ADRC algorithm based on NESO.

**Fig. 11:**
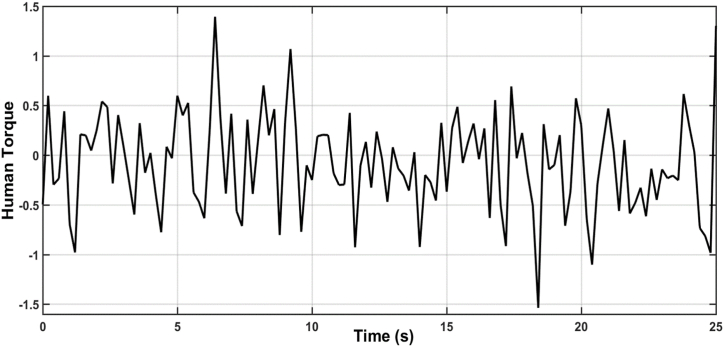
Uncertainty behaviour due to Human Torque.

**Fig. 12:**
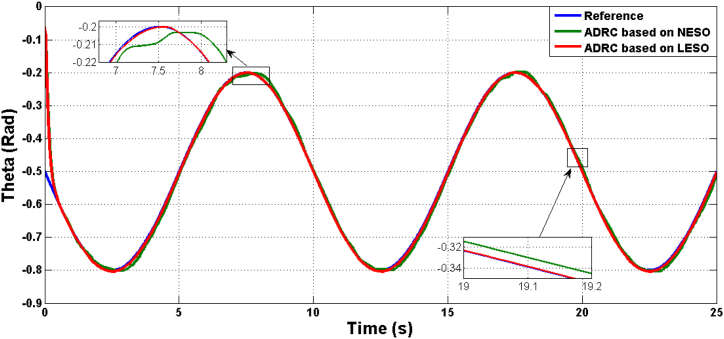
The responses of joint angle due to ADRC algorithm based on LESO and NESO in the uncertainty case.

**Fig. 13:**
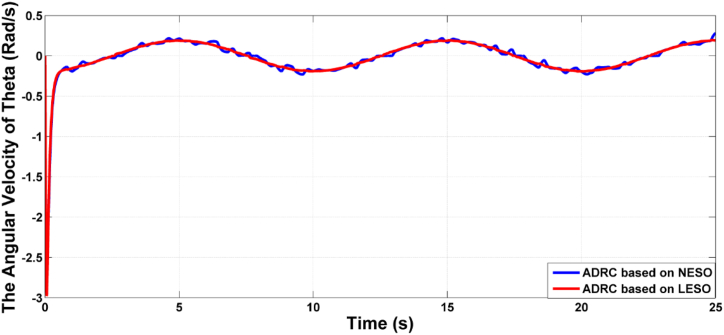
The rate change responses of joint angle of controlled system based on ADRCs with LESO and NESO in the uncertainty case.

**Fig. 14:**
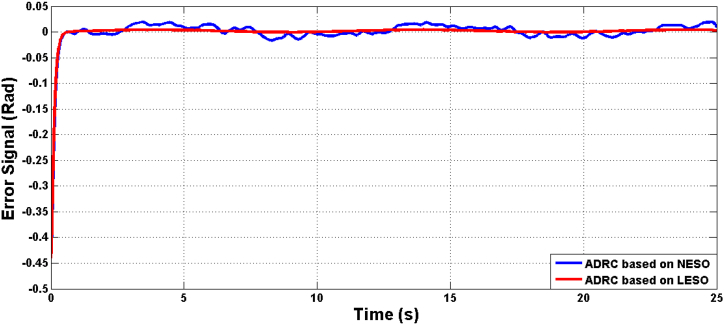
The error signal due to ADRC based on LESO and NESO in the uncertainty case.

**Fig. 15:**
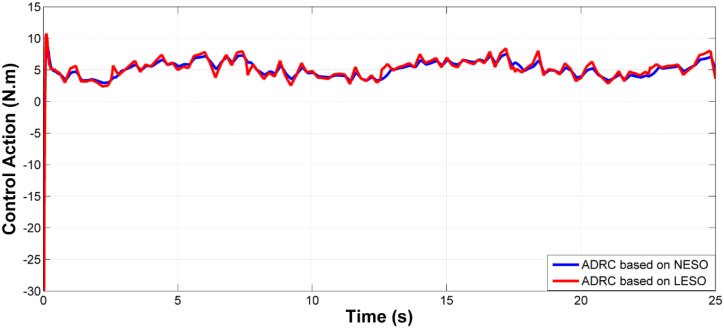
The control effort of controlled system based on ADRC with LESO and NESO in the uncertainty case.

Fig. 9, Fig. 10 illustrate the estimation of the system dynamics using both proposed observers. It can be inferred from these figures that the ADRC algorithm based on LESO outperforms the one based on NESO due to superior estimation capability. LESO provides more accurate estimations of the theta angle, rate of change of the theta angle, and system dynamics when compared to NESO. Finally, Fig. 11 showcases the human torque signal.In the subsequent scenario, the simulation is repeated under the presence of parameter uncertainties, where the parameters of the system deviate by 20% from their nominal values. Fig. 12, Fig. 13 display the responses of the joint angle and its derivative, respectively, for the controlled system using ADRC with LESO and NESO. The error signal is shown in Fig. 14. Additionally, Fig. 15 illustrates the control signals generated by ADRC using both observers.

**Fig. 16:**
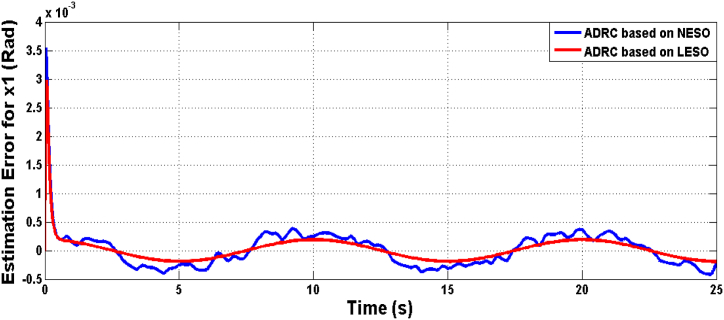
The estimation error of theta angle $x_{1}$ due to ADRC based on LESO and NESO subjected to uncertainty.

**Fig. 17:**
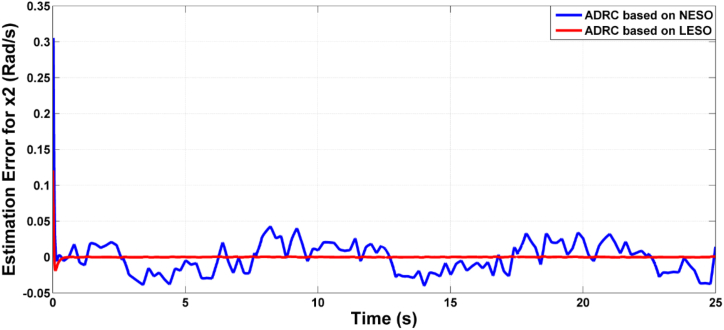
The estimation error of rate of joint angle $x_{2}$ due to ADRC based on LESO and NESO subjected to uncertainty.

**Fig. 18:**
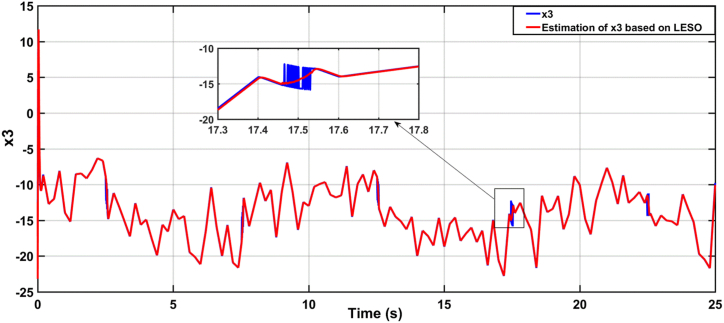
The estimation dynamics system due to ADRC algorithm based on LESO in the uncertainty case.

**Fig. 19:**
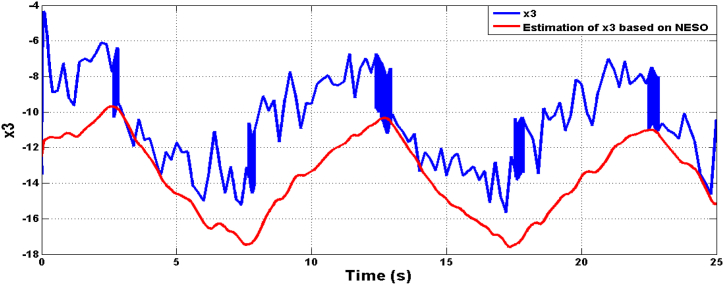
The estimation dynamics system due to ADRC algorithm based on NESO in the uncertainty case.

The estimation errors of LESO and NESO for state $x_{1}$ (angular position) and $x_{2}$ (rate of change of joint angle) is shown in Fig. 16, Fig. 17, respectively. The performance of LESO and NESO for estimating the uncertainty has been presented by Fig. 18, Fig. 19, respectively. The proposed controllers’ performance under the uncertainty scenario were assessed as shown in Table 3.

**Table 3:** Transient Parameters of Controlled System with ADRC Algorithm using LESO and NESO under Uncertainty.

Controller Type	RMSE	ISE	IAE	ITAE
ADRC algorithm based on LESO	0.8010	15.816	111.554	697.329
ADRC algorithm based on NESO	0.8787	19.036	258.806	2549.505

Results showed the efficiency of optimal LESO-based ADRC controller as comparison with optimal NESO-based ADRC controller. This competence is the result of the ability shown by the estimator LESO to accurately estimate the states $x_{1}$, $x_{2}$ and $x_{3}$.

## 5 Conclusion

In this study, the development of ADRCs based on LESO and NESO for controlling the Exoskeleton-Knee Assistive system was performed. The design parameters of both controllers were optimized using the Particle Swarm Optimization (PSO) algorithm to achieve optimal performance. The performance of the optimized LESO based ADRC was compared to that NESO based ADRC through computer simulations conducted in the MATLAB environment. The simulation results demonstrated the superiority of the LESO based ADRC algorithm in terms of its ability to accurately estimate the theta angle, the rate of change of the theta angle, and the dynamic system compared to the NESO based ADRC. Moreover, based on numerical results, it has been shown that the optimal ADRC based on LESO gives better performance as compared to optimal ADRC-based on NESO in terms of steady-state and transient characteristics. One can extend the proposed study for future work by suggesting other optimization methods like SSO (Spider Social Optimization), differential evolutionary technique, GA (Genetic Algorithm), and GWO (grey-wolf optimization) [[40], [41], [42]]. Moreover, a comparison study can be conducted between the proposed observer-based ADRC and other recent control structures [[43], [44], [45], [46], [47], [48], [49]]. Finally, the rehabilitation control techniques can be updated by including recent artificial intelligent approaches [[50], [51], [52]].

## Data availability

The data that support the findings of this study are available from the corresponding author, [A. Q. Al-Dujaili], upon reasonable request.

## CRediT authorship contribution statement

**Ayad Q. Al-Dujailii:** Writing – review & editing, Validation, Project administration, Funding acquisition. **Alaq F. Hasan:** Software, Investigation, Formal analysis, Conceptualization. **Amjad J. Humaidi:** Writing – original draft, Supervision, Methodology, Data curation. **Ammar Al-Jodah:** Visualization, Software, Resources, Formal analysis.

## Declaration of competing interest

The authors declare that they have no known competing financial interests or personal relationships that could have appeared to influence the work reported in this paper.
